# TSLP promoting B cell proliferation and polarizing follicular helper T cell as a therapeutic target in IgG4-related disease

**DOI:** 10.1186/s12967-022-03606-1

**Published:** 2022-09-08

**Authors:** Hui Lu, Xunyao Wu, Yu Peng, Ruijie Sun, Yuxue Nie, Jingna Li, Mu Wang, Yaping Luo, Linyi Peng, Yunyun Fei, Jiaxin Zhou, Wen Zhang, Xiaofeng Zeng

**Affiliations:** 1 Department of Rheumatology and Clinical Immunology, Peking Union Medical College Hospital, National Clinical Research Center for Dermatologic and Immunologic Diseases (NCRC-DID), State Key Laboratory of Complex Severe and Rare Diseases, Chinese Academy of Medical Sciences & Peking Union Medical College, Beijing, China; 2 Department of Stomatology, Peking Union Medical College Hospital, Chinese Academy of Medical Sciences & Peking Union Medical College, Beijing, China; 3 Department of Nuclear Medicine, Peking Union Medical College Hospital, Chinese Academy of Medical Sciences & Peking Union Medical College, Beijing, China

**Keywords:** IgG4-related disease, Thymic stromal lymphopoietin, B cell, Follicular helper cell, JAK-STAT

## Abstract

**Objective:**

To figure out the functions of thymic stromal lymphopoietin (TSLP) in IgG4-related disease (IgG4-RD).

**Methods:**

Plasma TSLP levels were tested by Elisa, and its receptors were detected by flow cytometry. Expressions of TSLP and TSLPR in involved tissues were stained by immunohistochemistry and immunofluorescence. Proliferation, apoptosis, and B subsets of TSLP stimulated-B cells were analyzed by flow cytometry. TSLP-stimulated B cells were co-cultured with CD4+ Naïve T cells. Signaling pathway was identified by RNA-sequencing and western blot. Anti-TSLP therapy was adapted in Lat^Y136F^ knock-in mice (Lat, IgG4-RD mouse model).

**Results:**

Plasma TSLP level was increased in IgG4-RD patients and was positively correlated with serum IgG4 level and responder index (RI). TSLPR was co-localized with CD19+ B cells in the submandibular glands (SMGs) of IgG4-RD. TSLP promoted B cell proliferation, and TSLP-activated B cells polarized CD4+ naive T cells into follicular helper T (Tfh) cells through OX40L. RNA-sequencing identified JAK-STAT signaling pathway in TSLP-activated B cells and it was verified by western blot. Anti-TSLP therapy alleviated the inflammation of lung in Lat mice.

**Conclusion:**

Elevated TSLP in IgG4-RD promoted B cells proliferation and polarized Tfh cells and might be served as a potential therapeutic target.

**Supplementary Information:**

The online version contains supplementary material available at 10.1186/s12967-022-03606-1.

## Introduction

IgG4-related disease (IgG4-RD) is an immune-mediated fibroinflammatory disease characterized by elevation of serum IgG4 level and infiltration of IgG4+ plasma cells in the involved organs [1–4]. The predominant features in the pathophysiology of IgG4-RD are oligoclonal expansion of plasmablasts and CD4+ T follicular helper cells (Tfh) expansion [5, 6].

Thymic stromal lymphopoietin (TSLP), initially described as a lymphocyte growth factor, belongs to T helper 2 (Th2) cytokines [7, 8], and its two receptors are TSLPR (also known as CRLF2) and interleukin (IL)-7Ra (CD127, shared with IL-7) [9]. TSLP stimulated-DC could specifically induce Th2 induction, which has been widely described in allergic diseases. For instance, DC stimulated with TSLP could secrete Th2-attracting chemokines such as thymus and activation regulated chemokine (TARC) and macrophage derived chemokine (MDC), and induce the production of Th2 cytokines by CD4+ T cells, including IL-4, IL-5 and IL-13 [9, 10]. Besides, TSLPR signaling could also negatively regulate IL-1β production in DC [11], and TSLP could enhance group 2 innate lymphoid cell (ILC2) activation, synergistically with IL-33 [12]. TSLP was also reported to upregulate the expression of collagen I and a-SMA in fibroblast, aggravating asthmatic airway remolding [13].

Recently, TSLP was implicated as a disease exacerbating mediator in autoimmune diseases. TSLP was reported to overexpressed in the skin samples of psoriasis, systemic sclerosis (SSc), and synovial fluid of rheumatoid arthritis (RA) [14–16]. In primary Sjogren syndrome (pSS), TSLP was located with B cells in the submandibular glands (SMGs) and progressively increased from benign to malignant B cell lymphoproliferation [17]. Yajima et al. [18] found TSLP was upregulated in the SMGs of patients with IgG4-RD, but failed to clarify the exact role of TSLP in IgG4-RD.

Independent evidences indicate TSLP a strong candidate of pathogenic factors in the pathogenesis of IgG4-RD. First, TSLP is a member of Th2 cytokines family and IgG4-RD is considered to be a Th2 predominant disease [19]. Second, TSLP is reported to be associated with allergy and IgE secretion [7] and IgG4-RD patients are frequently suffered from allergic conditions [20], and elevated serum IgE are commonly observed in IgG4-RD. Third, Tfh expansion is one of the significant characteristics of IgG4-RD, but the Th2 cytokines like IL-4, inhibit the differentiation of Tfh. TSLP was reported to activate DC to promote Tfh polarization [21]. Therefore, in this study, we focus on the expression and the role of TSLP in the pathogenesis of IgG4-RD.

## Method

### Patients

Seventy-one newly diagnosed and treatment naïve patients with IgG4-RD fulfilling 2019 classification criteria of American College of Rheumatology (ACR)/European League Against Rheumatism (EULAR) for IgG4-RD [22] were enrolled in this study, and patients with current infections, malignances, other autoimmune diseases, and conditions that could mimic IgG4-RD were all excluded. Details of clinical characteristics of IgG4-RD patients were shown in Additional file 2: Table S1. Besides, forty-one age and sex matched health controls (HCs) were enrolled. Disease activity was assessed by IgG4-RD responder index (RI) [23]. Remission was defined as IgG4-RD RI of every single organ ≤ 1 and glucocorticoid tapered to maintenance dosage without relapse. Forty IgG4-RD patients with disease remission were also included in this study. In addition, six SMG samples of treatment naïve IgG4-RD patients and three labial gland samples from patients with untreated pSS were obtained.

This study was approved by the ethnic committee of Peking Union Medical College Hospital (approval number, S-442) and written informed consents were obtained from all patients and healthy volunteers.

### Animals

Lat^*Y136F*^ knock-in mice (Lat) were obtained from Institute of laboratory animal Sciences, Chinese Academy of Medical Sciences & Peking Union Medical College, according to the previous literature [24]. Lat mice (on a C57BL/6 background) and wild type C57BL/6 (WT) mice were maintained in specific pathogen free (SPF) conditions. Lat mice were screened by PCR, and the following primers were used: 5′-TAGGCTACCTGTGAGTGGGTAG-3′ (forward), 5′-AAACCTACCCACTCACAGGTAG-3′ (reverse). Cervical dislocation was performed to sacrifice mice. Lat mice (n = 25, male 13, female 12) and WT (n = 14, male 7, female 7) were sacrificed at ages of 8 weeks or 12 weeks. Blood was collected from the eyeball, and the SMGs, pancreas, kidneys, liver, spleen, and lung were obtained. All animal experiments were approved by ethnic committee of Peking Union Medical College Hospital (approval number, XHDW-2020-027).

### Enzyme-linked immunoassay (Elisa)

The plasma of humans and mice were collected and stored at − 80 °C until used. The concentrations of TSLP in human (Cloud-Clone) and mouse (CUSABIO) were measured by Elisa kit. Antibodies in B cell culture supernatant was quantified by IgG Elisa kit (CUSABIO), IgG4 Elisa kit (CUSABIO) and IgE Elisa kit (Bethyl Laboratories).

### Histology, immunohistochemistry and immunofluorescence

Formalin-fixed, paraffin-embedded samples were cut into consecutive 3-μm thick sections. Mouse samples were also stained with hematoxylin–eosin (HE) for inflammation observation and Masson for fibrosis assessment. The degree of inflammation and fibrosis were determined as the ratio of the inflammatory or fibrotic area to the whole stained area in a 4 μm^2^ field of view from 5 different areas.

The primary antibodies were: anti-human TSLP, anti-mouse IgG1, anti-human CRLF2 (TSLPR) from abcam. Anti-mouse TSLP, anti-human CD19, anti-human CD11c, anti-human CD4, anti-mouse CRLF2, and anti-mouse B220 (CD45) from Servicebio. DAPI was used to stain nuclei. Immunofluorescence images were acquired and merged by EVOS FL Auto 2 Imaging System (ThermoFisher Scientific).

### Flow cytometry

The antibodies were all from biolegend: anti-CD19, anti-CD3, anti-CD4, anti-CD8, anti-Lineage (Lin), anti-HLA-DR, anti-CD11c, anti-TSLPR, anti-IL-7Ra, anti-CD45RA, anti-CD62L, anti-Ki-67, anti-CD24, anti-IgD, anti-CD27, anti-CD38, anti-CXCR5, anti-PD-1, anti-CD25, anti-foxp-3; anti-IL-4, anti-OX40L, anti-PD-L1, anti-CD80, anti-CD86, anti-ICOSL, and isotype-matched controls. For apoptosis analysis, PE Annexin V Apoptosis Detection Kit I (BD Biosciences) was used. All experiments were measured by BD FACSAria II system (BD Biosciences) and data were analyzed by Flowjo version X software (Flowjo, Ashland, OR, USA).

### Cell isolation

Peripheral blood mononuclear cells (PBMCs) were isolated by standard Ficoll-Hypaque procedures. CD19+ B cells and CD4+ Naïve T cells were both enriched by negative selection with isolation kits (Miltenyi Biotec). The purity of B cells or Naïve T cells was determined by flow cytometry to obtain 98% purity (Additional file 1: Fig S1).

### Proliferation, apoptosis, and differentiation, antibody measurement of B cells

Cells were cultured in complete RPMI 1640 medium supplemented with 10% fetal calf serum and 100 U/ml Penicillin/Streptomycin (Gibco). B cells were activated in the presence of 50 ng/ml TSLP (peprotech) or PBS, 10 µg/ml anti-human IgM (Invitrogen), 500 ng/ml recombinant human CD40L (Abcam), 100 ng/ml IL-4 (peprotech). B cells were collected on day 5 for proliferation, apoptosis, differentiation analysis by flow cytometry. Besides, the supernatant was harvested on day 7 and IgG, IgG4 and IgE were quantified by Elisa.

### Cell coculture

For co-culture, B cells were cultured in the presence of 50 ng/ml TSLP or PBS for 72 h. Then, TSLP-B or B cells were washed two times and put in culture with CD4+ Naïve T cells that freshly purified from healthy donors (5 × 10^4^ B cells and 5 × 10^4^ Naïve T cells) in 96-well round-bottom plates in the presence of 5 µg/ml purified plate-bound anti-CD3 mAb (BD Biosciences), 5 µg/ml purified anti-CD28 mAb (BD Biosciences) for 5 days.

For transwell experiments, B cells and CD4+ Naïve T cells were seeded in the upper and lower chambers respectively, with a 0.4-µm polycarbonate semipermeable membrane (Corning-Costar). To detect the costimulatory molecules on B cells, B cells were cultured with 50 ng/ml TSLP or PBS, combined with 10 µg/ml anti-human IgM, 500 ng/ml recombinant human CD40L for 72 h. For blocking experiments, anti-human OX40L antibody (abcam) or control antibody were added to the culture.

### RNA-sequencing

B cells were stimulated with TSLP or PBS for 72 h. Total RNA of TSLP-B and B cells were isolated using TRIZOL (Invitrogen Carlsbad, USA) and were quantified using the RNA Nano 6000 Assay Kit of the Bioanalyzer 2100 system (Agilent Technologies, CA, USA; Supplementary methods were shown in Additional file 4).

### Western blot

B cells were preactivated with anti-IgM, CD40L for 72 h, and then stimulated with TSLP or anti-TSLP antibody (proteintech) for 60 min. Total protein of B cells was extracted by Minute Total Protein Extraction Kit (Invent Biotechnologies, USA), and concentrations were determined by BCA Assay kit (Pierce Biotechnology, USA). Protease inhibitor cocktail (huaxingbio) and phosphatase inhibitor (Keygen Biotech) were added to the lysis buffer. Antibodies for immunoblotting: phosphorylation (p)-JAK1, JAK1, p-JAK2, JAK2, p-JAK3, JAK3, p-Stat1, Stat1, p-Stat3, Stat3, p-Stat5, Stat5, β-actin were all from Cell signaling Technology. Images were captured and analyzed on Chemiluminescent Imaging System. Total density of each protein band was determined and the ratio of target protein to β-actin density was calculated.

### Anti-TSLP therapy in mice

Lat mice of same litter were randomly assigned into two groups: anti-TSLP therapy group (n = 5) and phosphate-buffered saline (PBS) group (n = 5). 50ug anti-TSLP antibody (R&D systems) in 0.2 ml of PBS or PBS was administered intraperitoneally once a week starting from 4 weeks of age until 7 weeks in Lat mice. Mice were sacrificed at 8 weeks of age, and plasma and organs were obtained, respectively. The efficacy was measured by the degree of inflammation and fibrosis in affected organs of Lat mice.

### Statistical analysis

Statistical analysis was performed using the GraphPad Prism version 7 and IBM SPSS Statistics version 22. The t-test was used for parametric data and the Mann–Whitney U-test for non-parametric data between two groups. One-way analysis of variance or Kruskal–Wallis test was used when there were more than two groups, and followed by a post hoc Tukey test or Dunn’s test. Paired t test or Wilcoxon test were applied to compare two paired groups. Categorical parameters were assessed by Fisher’s exact test or chi-square test. Correlations between variables were analyzed by Pearson’s rank test (normally distributed data) or Spearman’s rank correlation test (non-normally distributed data). A two-tailed p-value < 0.05 was considered as statistically significant. Normally distributed data are shown as means ± standard error of mean (SEM), continuous non-normally distributed data are presented as median and interquartile range (IQR).

## Result

### The expression levels of TSLP and its receptors in IgG4-RD

**Fig. 1:**
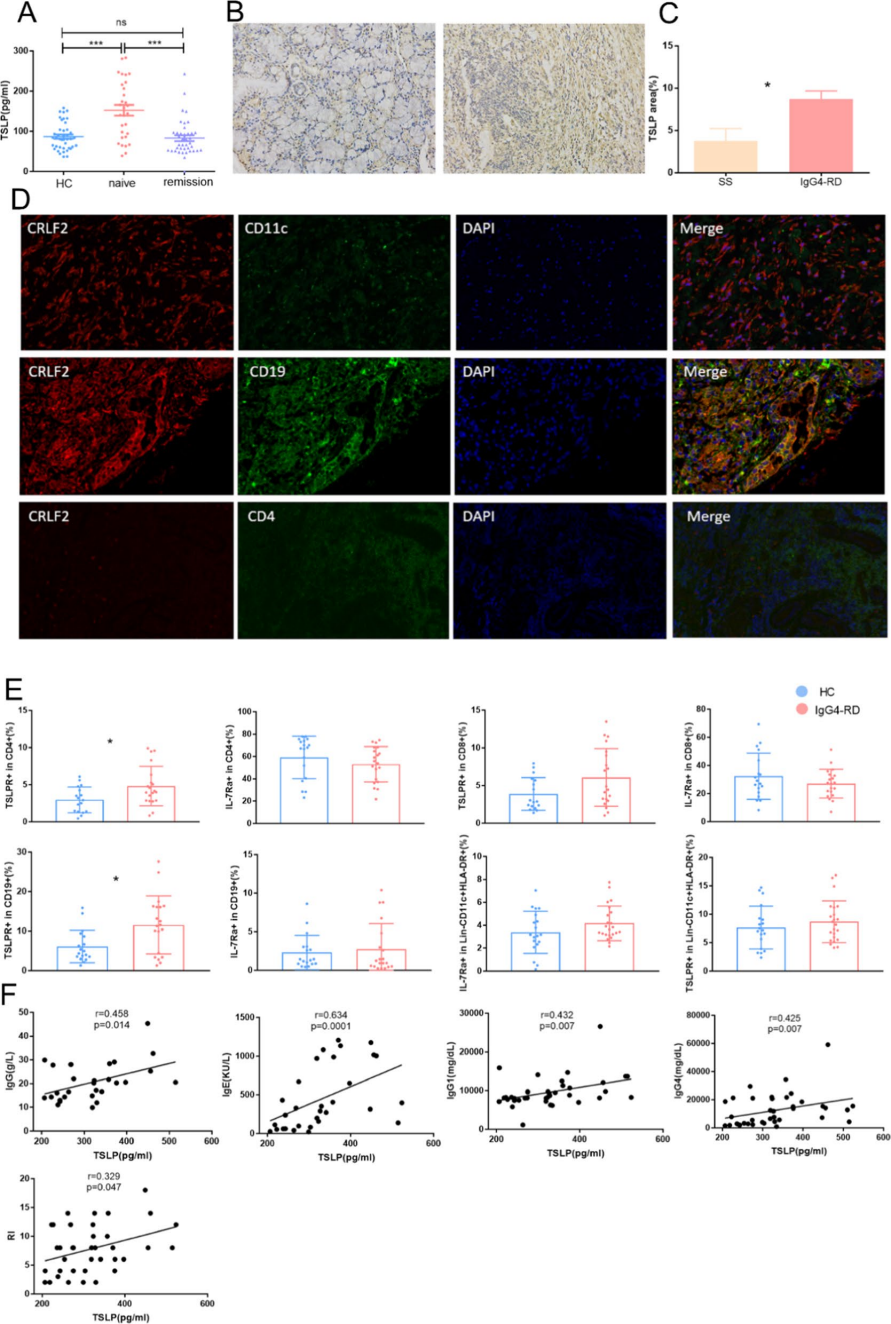
The expression of TSLP and its receptors in IgG4-RD. **A** Plasma levels of TSLP in HC, treatment naïve IgG4-RD patients and IgG4-RD patients with disease remission measured by Elisa. **B** Representative immunohistochemical staining of TSLP in LG from patients with SS (left) and SMG from patients with IgG4-RD (right). **C** The expression levels of TSLP in SMG estimated by semiquantitative analysis. **D** Trible staining for CD11c (green), CD4 (green), CD19 (green), CRLF2 (TSLPR, red) and DAPI (blue) in SMG from representative patients with IgG4-RD. **E** Percentages of TSLPR and IL-7Ra in CD4+, CD8+, CD19+, and Lin-CD11c+HLA-DR+ cells respectively. **F** Correlations between serum levels of IgG, IgE, IgG1, IgG4, RI and plasma level of TSLP. *HC* healthy controls, *IgG4-RD* IgG4-related disease, *LG* labial gland, *SMG* submandibular gland, *SS* primary Sjogren’s syndrome, *RI* responder index. *p < 0.05; ***p < 0.001; ns = not significant

Firstly, Elisa revealed that TSLP level was upregulated in the plasma of untreated IgG4-RD patients compared with HCs (152.5 ± 13.3 pg/ml vs. 86.9 ± 5.2 pg/ml, p < 0.0001), and decreased in IgG4-RD patients with disease remission compared with untreated patients (83.0 ± 7.1 pg/ml vs. 152.5 ± 13.3 pg/ml, p < 0.0001, Fig. 1A). However, we did not find any difference of TSLP levels between IgG4-RD patients with and without allergic history (Additional file 1: Fig. S2). In immunohistochemistry, we observed TSLP was diffusely located in SMGs of IgG4-RD, which was much more frequent than that in the labial gland of pSS (8.71% ± 0.97% vs. 3.75% ± 1.48%, p < 0.05, Fig. 1B, C). To clarify which cell express TSLPR, triple immunofluorescence staining was performed in SMGs of patients in IgG4-RD and revealed that TSLPR-positive cells mainly colocalized with CD19+ cells (Fig. 1D).

We further examined the expression of the two TSLP receptors in lymphocytes by flow cytometry and representative gating strategy was shown in Additional file 1: Fig. S3. Notably, the level of TSLPR was upregulated in CD4+ T cells (4.81% ± 0.60% vs. 2.97% ± 0.42%, p = 0.020) and CD19+ B cells (11.56% ± 1.64% vs. 6.12% ± 0.97%, p = 0.024, Fig. 1E) in IgG4-RD compared with HCs. Correlation analysis found TSLP level was positively correlated with serum IgG (r = 0.458, p = 0.014), IgE (r = 0.634, p = 0.0001), IgG1 (r = 0.432, p = 0.007), IgG4 levels (r = 0.425, p = 0.007), and RI (r = 0.329, p = 0.047) (Fig. 1F). But there was no correlation between TSLP level and other laboratory parameters (Additional file 1: Fig. S4).

### The effect of TSLP on the proliferation, apoptosis, and differentiation of B cells

**Fig. 2:**
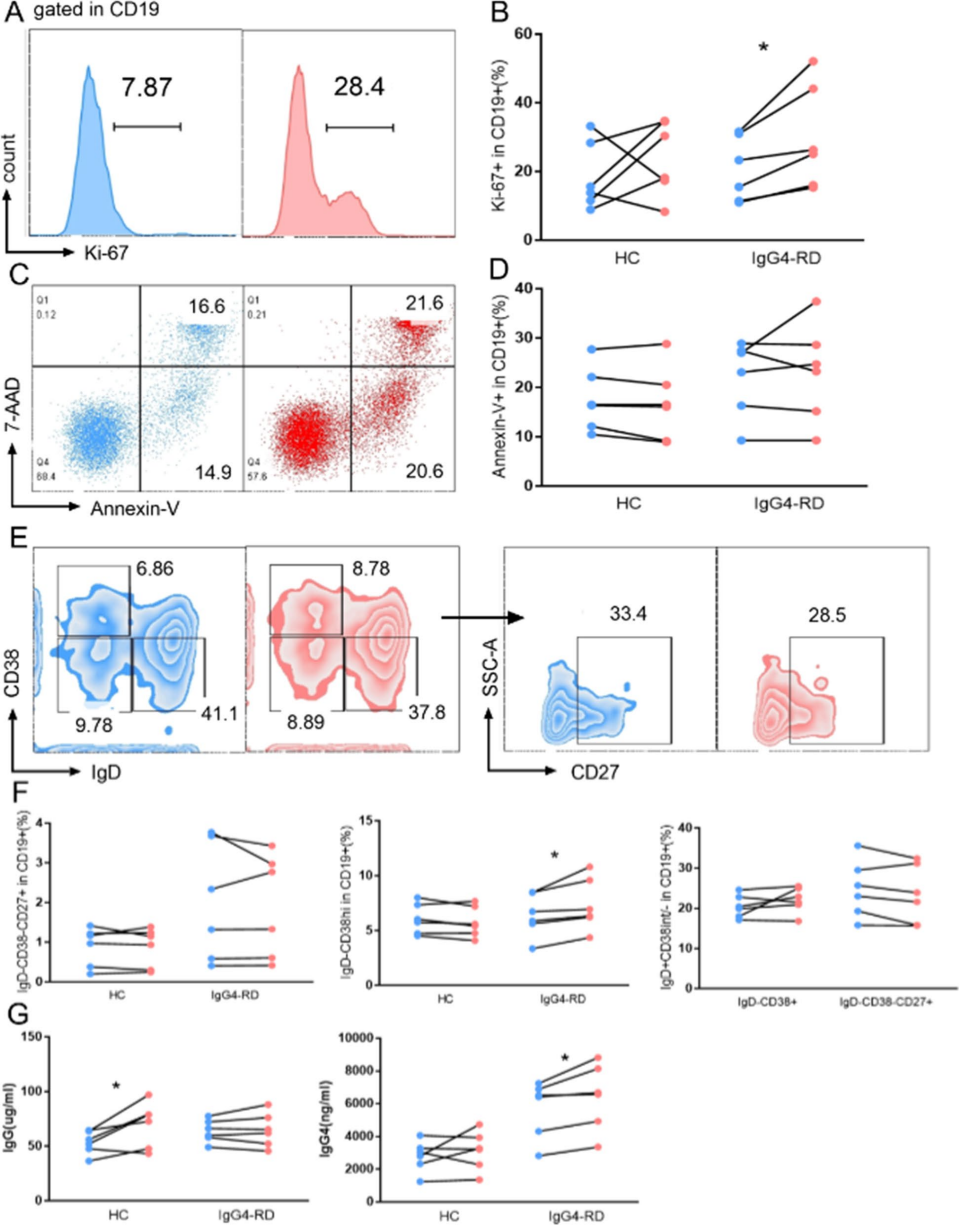
The effect of TSLP on B cells in IgG4-RD. CD19+ B cells from IgG4-RD patients were stimulated with IL-4, anti-IgM, CD40L, with or without TSLP. Proliferation quantified by Ki-67 (**A**, **B**), apoptosis measured by 7-AAD and Annexin-V (**C**, **D**), and B cell subsets (**E**, **F**) were assessed on 5 days. Supernatant IgG and IgG4 levels on 7 days were quantified by ELISA (**G**). *p < 0.05

Since plasma TSLP level was positively correlated with serum IgG, IgG1, IgG4, and IgE levels, and immunofluorescence revealed that CD19+ cells mainly expressed TSLPR in IgG4-RD tissues, we tended to elucidate the potential function of TSLP on B cells (Fig. 2). Briefly, we observed that TSLP significantly upregulated the expression of Ki-67 in IgG4-RD patients (29.8% ± 6.2% vs. 20.6% ± 3.8%, p = 0.031, Fig. 2A, B), while apoptosis was comparable between the two groups (Fig. 2C, D). Notably, B subsets were analyzed and we found TSLP upregulated the percentages of IgD-CD38hi plasmablast (7.39% ± 0.97% vs. 6.43% ± 0.79%, p = 0.031, Fig. 2E, F). In addition, supernatant IgG, IgG4, and IgE levels on 7 days were quantified by Elisa and revealed that TSLP upregulated IgG levels in HCs (69.7 ± 8.4 µg/ml vs. 53.4 ± 4.3 µg/ml, p = 0.035) as wells as IgG4 levels in IgG4-RD patients (6412.4 ± 824.2 ng/ml vs. 5696.2 ± 711.1 ng/ml, p = 0.031, Fig. 2G). IgE could not be detected in the supernatant.

### TSLP-activated B cells polarize naive CD4+ T cells into Tfh cells through OX40L

**Fig. 3:**
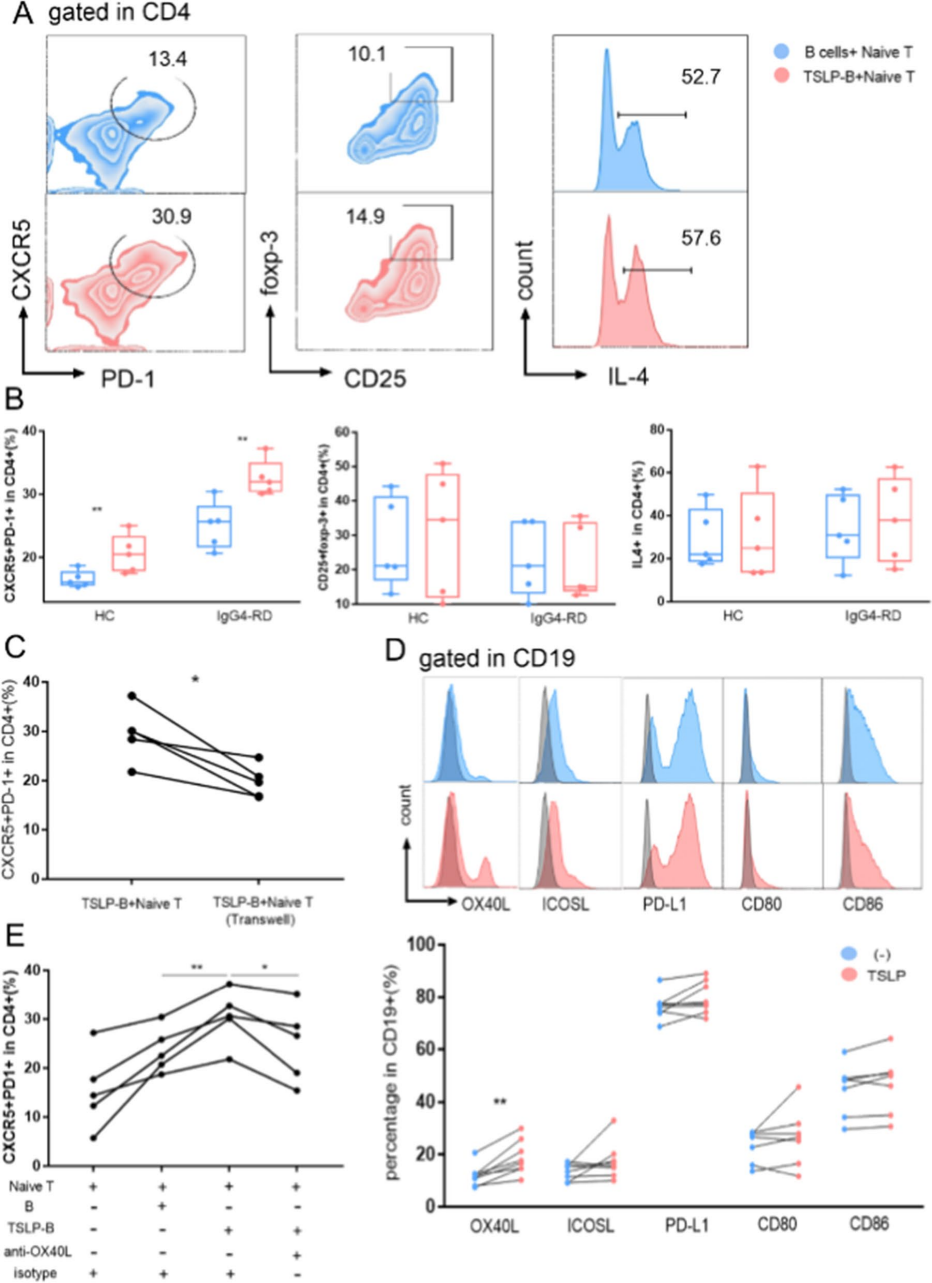
TSLP-activated B cells polarized Naïve T cells into Tfh cells though OX40L. CD19+ B cells from HC or IgG4-RD patients were stimulated with or without TSLP for 3 days and then were co-cultured with CD4+ Naïve T cells from HC with anti-CD3 and anti-CD28 for 5 days. Representative FACS (**A**) and summary graphs (**B**) of Tfh gated as CXCR5+PD-1+, Treg gated as CD25+foxp-3+, and IL-4+ in CD4+ T cells. **C** TSLP-B cells were directly or indirectly (transwell) co-cultured with CD4+ Naïve T cells, with anti-CD3 and anti-CD28 for 5 days and Tfh were measured by FACS. B cells were stimulated with anti-IgM, CD40L, with or without TSLP for 3 days, and representative FACS and summary graphs (**D**) showed the changes of co-stimulatory molecules on B cells. **E** B cells or TSLP-B cells were co-cultured with CD4+ Naïve T cells with anti-CD3, anti-CD28, anti-OX40L antibody or isotype control for 5 days and Tfh were measured by FACS. TSLP-B, TSLP activated B cells

Previous literatures have reported that TSLP contributed to Th2 and Tfh polarization through DC activation [8, 21, 25], and we tried to find out the effect of TSLP-stimulated B cells on the differentiation of CD4+ Naïve T cells. CD19+ B cells were stimulated with TSLP (TSLP-B), and then were put in culture with CD4+ Naïve T cells from HCs. To remove the intrinsic property of B cells, we used non-stimulated B cells from the same person as a negative control. After 5 days of co-culture, we detected a predominant Tfh polarization induced by TSLP-B in IgG4-RD patients (32.5% ± 1.3% vs. 25.0% ± 1.7%, p = 0.007, Fig. 3A, B), but not Treg or Th2 polarization. To check whether TSLP polarize Tfh directly, CD4+ Naïve T cell were stimulated with or without TSLP for 5 days, which showed TSLP could not induce Tfh differentiation directly (Additional file 1: Fig. S5).

To gain mechanistic insight into the polarization of Tfh, TSLP-B cells and CD4+ Naïve T cells were co-cultured in direct cell–cell contact group and indirect transwell culture group, which showed TSLP-B induced Tfh polarization more apparently in direct contact group (29.5% ± 2.5% vs. 19.8% ± 1.5%, p = 0.016, Fig. 3C). Hence, we focused on surface co-stimulatory molecules associated with Tfh differentiation and measured the expression of OX40L, ICOSL, PD-L1, CD80 and CD86 on B cells after stimulation of TSLP or PBS for 72 h by flow cytometry. We observed that TSLP-B expressed higher levels of OX40L (19.0% ± 2.3% vs. 12.0% ± 1.4%, p = 0.008, Fig. 3D). Next, we confirmed the role of OX40L by culturing TSLP-B with CD4+ Naïve T cells in the presence of anti-OX40L blocking antibody or control antibody for 5 days. As expected, we observed that OX40L functional blocking indeed decreased the Tfh expression (Fig. 3E). Meanwhile, we assessed the percentages of Tfh and the expression levels of costimulatory molecules on B cells in both HCs and IgG4-RD patients. We found the percentages of Tfh (9.50% ± 1.19% vs. 4.48% ± 0.41%, p < 0.001) as well as the expression levels of OX40L (21.8% ± 3.5% vs. 9.8% ± 1.0%, p = 0.002) and CD86 (21.3% ± 4.0% vs. 11.7% ± 1.9%, p = 0.044) were increased in IgG4-RD patients (Additional file 1: Fig. S6). Furthermore, there was a positive correlation between the percentages of Tfh and the expression level of OX40L on B cells in IgG4-RD patients (r = 0.528, p = 0.036), and the percentages of Tfh was positively correlated with the level of CD86 on B cells in HCs (r = 0.685, p = 0.007, Additional file 1: Fig. S6).

### TSLP activated B cells via JAK-STAT signaling pathway

**Fig. 4:**
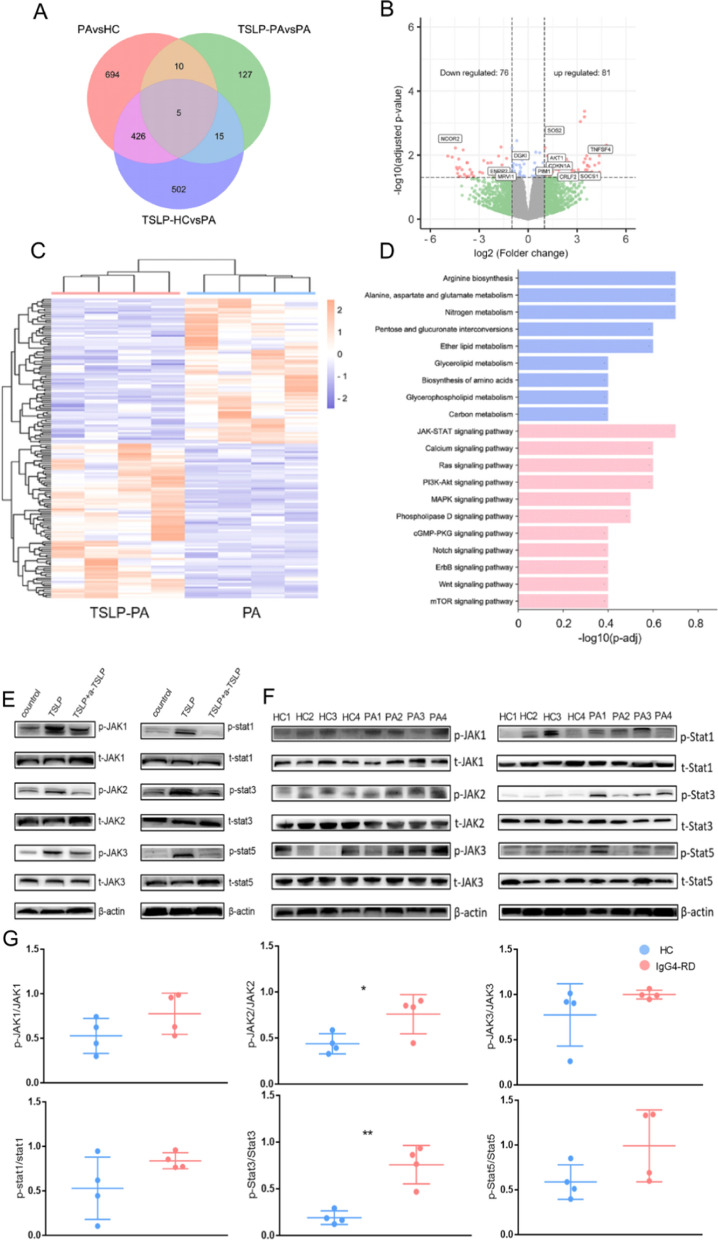
TSLP activated JAK-STAT signaling pathway of B cells in IgG4-RD. CD19+ B cells from HC and IgG4-RD patients were stimulated with or without TSLP for 3 days and were detected by RNA-sequencing. **A** Venn diagram showed overlap of differential expression genes (DEGs) compared between TSLP-B and B cells from IgG4-RD patients (PA) and HC. **B** Volcano plot of DEGs of TSLP-B cells (n = 4) and B cells (n = 4) from IgG4-RD patients showed downregulated genes (left, n = 76), and upregulated genes (right, n = 81). **C** Heatmap of DEGs (n = 157) between TSLP-B and B cells from IgG4-RD patients. **D** KEGG enrichment of DEGs in metabolism (blue) and environmental information processing (red) of TSLP-B and B cells from IgG4-RD patients. **E** CD19+ B cells from HC were activated with anti-IgM, CD40L for 72 h, then were stimulated with or without TSLP, with or without anti-TSLP antibody for 60 min. Activated JAK-STAT family molecules were detected by western blotting with β-actin serving as an internal control. **F**, **G** CD19+ B cells from HC or IgG4-RD patients were activated with anti-IgM, CD40L for 72 h, and representative western blot (**F**) and summary graphs (**G**) of phosphorylated forms of JAK-STAT were shown

B cells sorted from HCs or patients with IgG4-RD were stimulated with or without TSLP, and whole transcriptome sequencing was performed. Differential expression genes (DEGs) were shown in Fig. 4A, C. Totally, 157 DEGs (upregulated n = 81, downregulated n = 76) were identified between B cells and TSLP-B cells from IgG4-RD patients (Fig. 4B). Details of DEGs was shown in Additional file 3: Table S2. Kyoto Encyclopedia of Genes and Genomes (KEGG) enrich analysis revealed metabolic pathways and inflammation pathways in TSLP-stimulated B cells (Fig. 4D). Of which, we focused on JAK-STAT signaling pathway that was widely reported to be relevant with TSLP. Phosphorylation of JAK-STAT family was detected after the stimulation of TSLP for 0, 10, 30, 60, 90 min (Additional file 1: Fig. S7). Activated of JAK-STAT family were also analyzed in B cells with anti-TSLP antibody (Fig. 4E). Consistently, western bolt analysis revealed the phosphorylation of JAK2 (0.76 ± 0.11 vs. 0.44 ± 0.06, p = 0.037) and Stat3 (0.76 ± 0.10 vs. 0.19 ± 0.04, p = 0.002) were upregulated in the B cells of IgG4-RD patients compared with HCs (Fig. 4F, G).

### The expression of TSLP and the efficacy of anti-TSLP antibody in Lat mice

Lat mice was featured as inflammatory mononuclear cell infiltration and fibrosis in multi-organs such as salivary gland, pancreas, as well as excellent responsibility to glucocorticoid therapy, which was considered to be the most appropriate experimental model for human IgG4-RD so far and it was recognized and selected for vivo experiments in a growing number of studies [26–29]. WT and Lat mice were sacrificed at age of 12 weeks and organs were obtained. Weight and imaging findings of Lat mice were shown in Additional file 1: Fig. S8. Briefly, PET-CT showed elevated uptake of salivary glands, lung, liver, and kidneys in Lat mice. IgG1 (a homologue of human IgG4) positive cells were observed in the SMGs, pancreas, lung and kidneys by immunohistochemistry staining (Additional file 1: Fig. S9).

In addition, Elisa detection showed the TSLP levels were upregulated in the plasma of Lat mice (Additional file 1: Fig. S8). Furthermore, immunohistochemistry displayed TSLP diffusely located in the organs and immunofluorescence staining for TSLPR (CRLF2, red) and CD19 (green) showed the colocalization of TSLPR and CD19+ cells were much more obvious in Lat mice than WT (Additional file 1: Fig. S10).

**Fig. 5:**
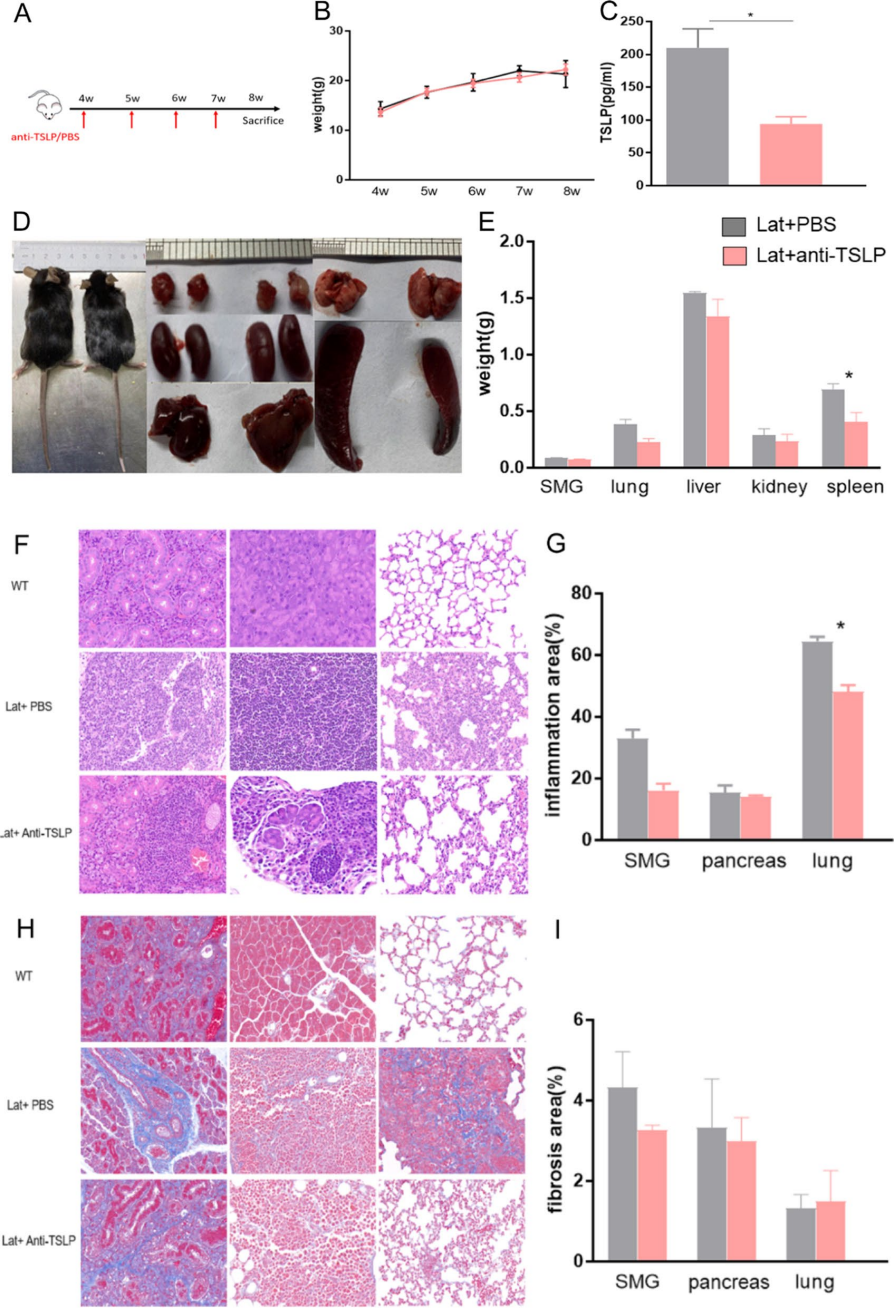
Efficacy of anti-TSLP antibody in Lat mice. **A** Lat mice was administered 50 µg anti-TSLP antibody (n = 5) in 0.2 mL of PBS or PBS (n = 5) intraperitoneally once a week starting from 4 weeks of age until 7 weeks, and were sacrificed at 8 weeks of age. Weight changes from 4 to 8 weeks (**B**), plasma TSLP levels at 8 weeks (**C**), morphology (**D**) and weight of submandibular glands (SMGs), lung, liver, kidneys, and spleen in Lat mice with PBS (left) and Lat mice with anti-TSLP therapy (right). Representative H&E-stained sections (×100) (**F**), comparison of inflammatory area (**G**), representative Masson’s trichrome stained sections (×100) (**H**), and comparison of fibrosis area (**I**) of SMGs, pancreas, lung from WT mice, Lat mice with PBS and anti-TSLP therapy

Based on the developing regularity of disease in Lat mice, and administration of anti-TSLP antibody reported in previous literature, intraperitoneal injection of anti-TSLP antibody was performed in Lat mice once a week from 3 weeks old, for a total of four times [12, 24, 30]. The representative inflammation area in the lung of Lat mice received with different dosage of anti-TSLP antibody was shown in Additional file 1: Fig. S11. The dosage of 50 µg anti-TSLP antibody was finally chosen and applicated in Lat mice. Briefly, Lat mice was administered with anti-TSLP antibody (50ug) or PBS intraperitoneally once a week from 4 weeks old to 7 weeks old, and were sacrificed at 8 weeks old (Fig. 5A). Changes of body weight over time was shown in Fig. 5B. The morphology, and comparison of the affected organs of Lat mice with anti-TSLP antibody therapy or PBS were shown in Fig. 5D, E. Representative HE staining and Masson staining were shown in Fig. 5F, H. We found anti-TSLP therapy downregulated the plasma TSLP levels (94.0 ± 11.2 pg/ml vs. 210.1 ± 29.0 pg/ml, p = 0.02, Fig. 5C), as well as alleviated the inflammation of lung (50.5% ± 3.0% vs. 62.3% ± 3.6%, p = 0.037, Fig. 5G), but had no influence on fibrosis in Lat mice (Fig. 5I).

## Discussion

**Fig. 6:**
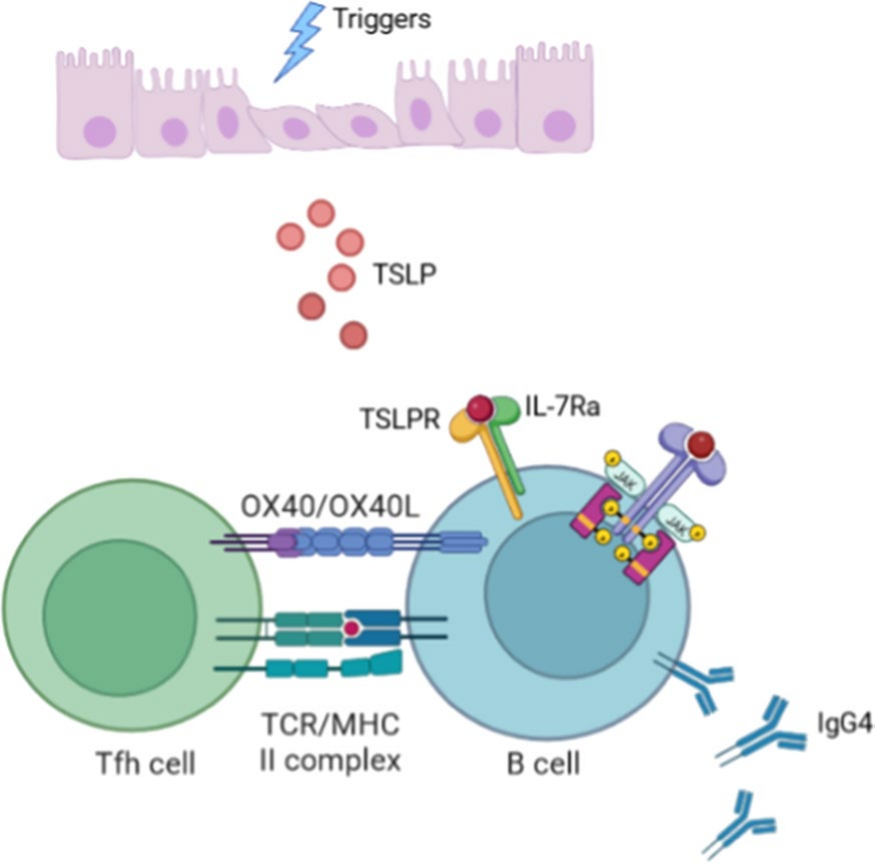
Schematic diagram of TSLP in the initiation of IgG4-RD. Unknown triggers promote epithelial cells or fibroblasts to release TSLP. TSLP binds to the receptors TSLPR and IL7Ra on B cells, activating JAK-STAT signaling pathway and encouraging the proliferation and IgG4 antibody secretion of B cells. Activated B cells upregulated the expression of OX40L and enhance the differentiation of Tfh cells

Here we showed for the first time, TSLP level was increased in the plasma of treatment naïve IgG4-RD patients and decreased in patients with disease remission. Besides, TSLP was also upregulated in the affected tissues of IgG4-RD, which was in lined with a previous study [18], and TSLPR was mainly colocalized with CD19+ B cells in SMGs of IgG4-RD. In addition, plasma TSLP level was positively correlated with serum antibodies such as IgG, IgE, IgG1, and IgG4 levels, as well as disease activity, which implicated that TSLP might participate in the pathogenesis of IgG4-RD by promoting immunoglobulin production of B cells (Fig. 6). We further presented evidence that TSLP directly enhanced the proliferation and expression of IgD-CD38hi plasmablasts in B cells. These were consistent with data published in healthy donors, TSLP was reported to support the production and proliferation of human B cell precursors [31, 32].

It was widely described that IL-12, IL-27, TGF-β could induce Tfh development [33–35]. IgG4-RD was reported to be a Th2 predominant disease, accompanied with a notable feature of Tfh expansion, but how Tfh polarization occur in Th2-environment remained unknown. TSLP was observed to possess the function of contributing to Th2 or Tfh polarization though DC [10, 21]. Instead of DC, we focused on the TSLP contribution on Th differentiation through B cell activation because immunofluorescence revealed a colocalization of TSLPR with B cells. More importantly, B cells was also required for Tfh response, as B cells could induce Bcl-6 expression in CD4+ T cells to induce and sustain Tfh differentiation [36–40]. We observed an induction of Tfh by TSLP activated-B cells. Moreover, the effect of TSLP-B on naïve T cells was reduced with transwell separation, implicating additional role of cell contact in this specific condition. To determine the potential factors, we explored the contribution of surface costimulatory molecules associated with Tfh differentiation that reported before [25, 41]. We observed TSLP-B expressed high levels of OX40L, and OX40L inhibition significantly decreased the percentages of Tfh cells. In parallel with our observations, Pattarini et al. [21] established that OX40L on TSLP-DC was the main driver of IL-21, and BCL-6 expression in T cells. Moreover, Jacquemin et al. [25] proposed that OX40L expressed on APCs in the blood and inflammatory tissues of SLE patients encouraged Tfh aberration. Nevertheless, as OX40L functional blocking did not completely abolish Tfh generation, we could not exclude that other factors might also attribute to the induction of Tfh by TSLP-B.

Mechanistically, RNA sequencing revealed several signaling pathways involved in TSLP activated-B cells. Several studies reported that TSLP activated JAK-STAT pathway in Naïve T cells, fibroblasts, and mDC [42–44]. Consequently, we verified the activation of JAK-STAT pathway in TSLP-stimulated B cells, and confirmed the phosphorylation of JAK2 and STAT3 was significantly upregulated in B cells from IgG4-RD patients. Our data indicating that JAK inhibitors might be a potential approach to restore homeostasis of B cells in IgG4-RD.

The upregulated levels of TSLP in plasma, and evident colocalization of TSLPR and B cells in affected organs made it reasonable to verify the efficacy of anti-TSLP antibody in Lat mice. As expected, anti-TSLP alleviated the inflammatory lesions in lung, but did not relieve the fibrosis in involved organs regretfully. In bleomycin SSc mice model, TSLPR-deficient mice showed less fibrosis development [45]. In RA mice model, recombinant TSLP injection caused more tissue destruction, while administration of anti-TSLP antibodies or deficiency of TSLPR could ameliorate inflammation [46, 47]. Accordingly, our findings suggested that TSLP might be a candidate of therapeutic targets of IgG4-RD.

There were some limitations in our study. RNA-sequencing identified several metabolic pathways activated in TSLP-stimulated B cells, but we failed to further elaborate them due to limited resources. Additionally, Lat mice might not be the optimal and ideal mice model of IgG4-RD, and more suitable and reliable mice model is desired to be established in the future for confirmatory pathogenic vivo experiments. Furthermore, the effect of anti-TSLP was not so satisfying as it slightly relieved inflammatory lesion area in lung tissue, but not in other affected organs. The low efficacy of anti-TSLP was not well understood currently, and it might be due to the interactions and the shared receptor with IL-7, which indicated that targeting on TSLPR or IL7Ra might bring more beneficial effect [9].

## Conclusions

Elevated TSLP expression in IgG4-RD promoted B cells proliferation and activated JAK-STAT signaling pathway. TSLP-activated B cells polarized CD4+ naïve T cells into Tfh cells through OX40L. TSLP was implicated in the pathogenesis of IgG4-RD and might be served as a potential therapeutic target.

## Supplementary Information

**Additional file 1: Figure S1.** Purity of CD19+ B cells and CD4+ Naïve T cells. FACS file showed CD19+ B cells (A) and CD4+ naïve T cells (B) were sorted to obtain 98% purity. **Figure S2.** There was no significant difference of TSLP levels between IgG4-RD patients with and without allergic history. **Figure S3.** Gating strategy and representative FACS plots of TSLPR and IL-7Ra in CD19+ cells of HC and IgG4-RD patients. **Figure S4.** There was no correlation between plasma TSLP level and other laboratory parameters. **Figure S5.** TSLP could not polarize Naïve T cells to Tfh directly. **Figure S6.** The percentage of Tfh was positively correlated with the percentages of OX40L+ on B cells in the peripheral blood of patients with IgG4-RD. **Figure S7.** Representative western blot showed the phosphorylation of JAK-STAT family with different time. **Figure S8.** Weight, imaging findings and TSLP expression of Lat mice. **Figure S9.** Immunohistochemistry of IgG1 in affected organs of Lat mice. **Figure S10.** Expressions TSLP and TSLPR in affected organs of Lat mice. **Figure S11.** Application of different dosage of anti-TSLP antibody Lat mice.

**Additional file 2: Table S1.** Baseline demographic features, clinical characteristics, and laboratory parameters of 71 treatment naïve patients with IgG4-RD.

**Additional file 3: Table S2.** List of differential expression genes of TSLP activated B cells and B cells from IgG4-RD.

**Additional file 4:** Supplementary Methods.

## Data Availability

Data are available from the corresponding author upon reasonable request.

## Abbreviations

TSLP: Thymic stromal lymphopoietin
IgG4-RD: IgG4-related disease
Lat: Lat^*Y136F*^ knock-in
RI: Responder index
SMGs: Submandibular glands
Tfh: Follicular helper T
Th2: T helper 2
IL: Interleukin
TARC: Thymus and activation regulated chemokine
MDC: Macrophage derived chemokine
SSc: Systemic sclerosis
RA: Rheumatoid arthritis
pSS: Primary Sjogren syndrome
ACR: American College of Rheumatology
EULAR: European League Against Rheumatism
HCs: Health controls
WT: Wild type
SPF: Specific pathogen free
Elisa: Enzyme-linked immunoassay
HE: Hematoxylin–eosin
Lin: Lineage
PBMCs: Peripheral blood mononuclear cells
PBS: Phosphate-buffered saline
SEM: Standard error of mean
IQR: Interquartile range
DEGs: Differential expression genes
p: Phosphorylation
