# Supplementary material for: TSLP promoting B cell proliferation and polarizing follicular helper T cell as a therapeutic target in IgG4-related disease
Source: J Transl Med. 2022 Sep 8;20:414. doi: 10.1186/s12967-022-03606-1 (PMC9461269; doi:10.1186/s12967-022-03606-1)
Supplement: Supplementary file 1 — Additional file 1: Figure S1. Purity of CD19+ B cells and CD4+ Naïve T cells. FACS file showed CD19+ B cells (A) and CD4+ naïve T cells (B) were sorted to obtain 98% purity. Figure S2. There was no significant difference of TSLP levels between IgG4-RD patients with and without allergic history. Figure S3. Gating strategy and representative FACS plots of TSLPR and IL-7Ra in CD19+ cells of HC and IgG4-RD patients. Figure S4. There was no correlation between plasma TSLP level and other laboratory parameters. Figure S5. TSLP could not polarize Naïve T cells to Tfh directly. Figure S6. The percentage of Tfh was positively correlated with the percentages of OX40L+ on B cells in the peripheral blood of patients with IgG4-RD. Figure S7. Representative western blot showed the phosphorylation of JAK-STAT family with different time. Figure S8. Weight, imaging findings and TSLP expression of Lat mice. Figure S9. Immunohistochemistry of IgG1 in affected organs of Lat mice. Figure S10. Expressions TSLP and TSLPR in affected organs of Lat mice. Figure S11. Application of different dosage of anti-TSLP antibody Lat mice. [file 12967_2022_3606_MOESM1_ESM.docx]

Figure S1. Purity of CD19+ B cells and CD4+ Naïve T cells. FACS file showed CD19+ B cells (A) and CD4+ naïve T cells (B) were sorted to obtain 98% purity.


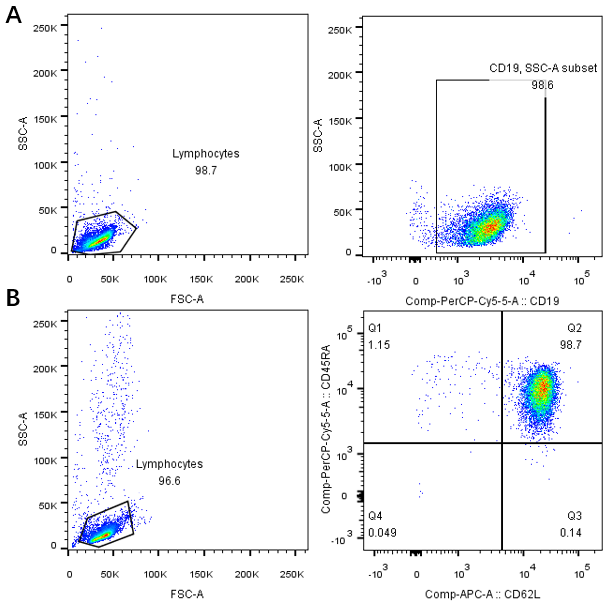


Figure S2. There was no significant difference of TSLP levels between IgG4-RD patients with and without allergic history.


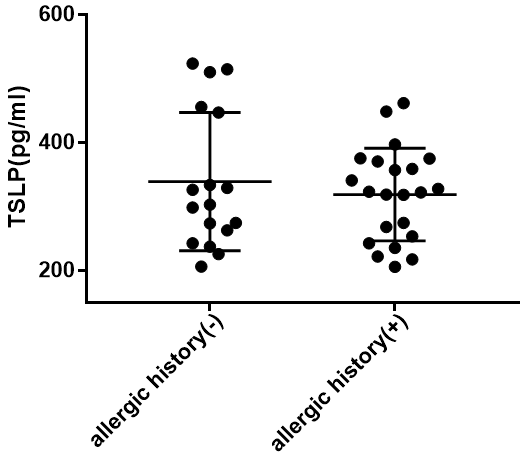


Figure S3. Gating strategy and representative FACS plots of TSLPR and IL-7Ra in CD19+ cells of HC and IgG4-RD patients.


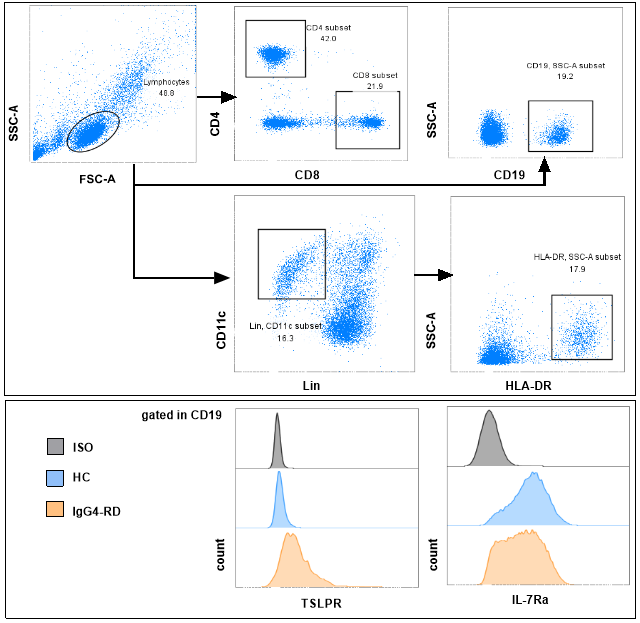


Figure S4. There was no correlation between plasma TSLP level and other laboratory parameters.


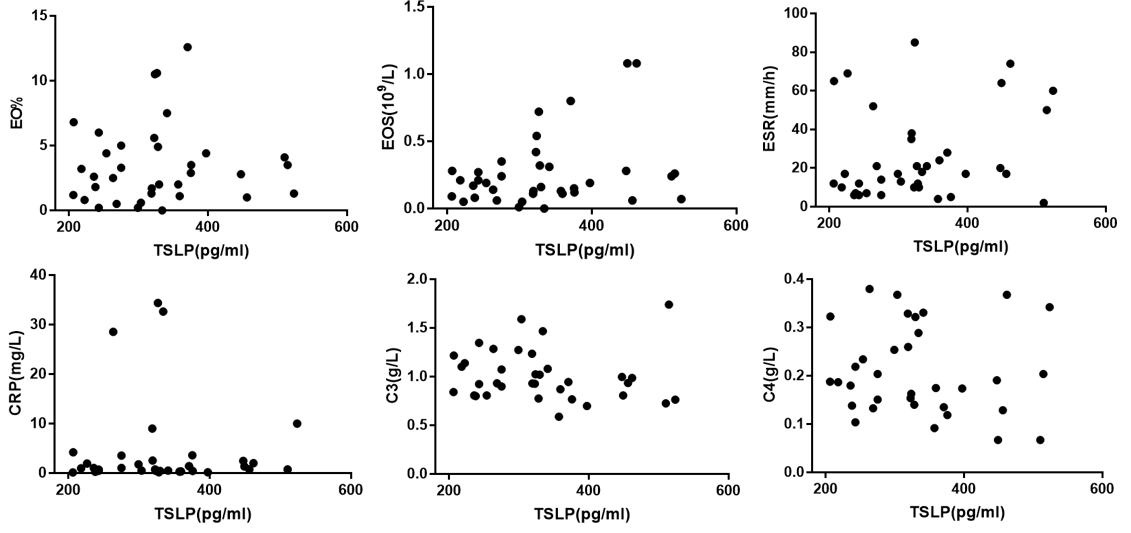


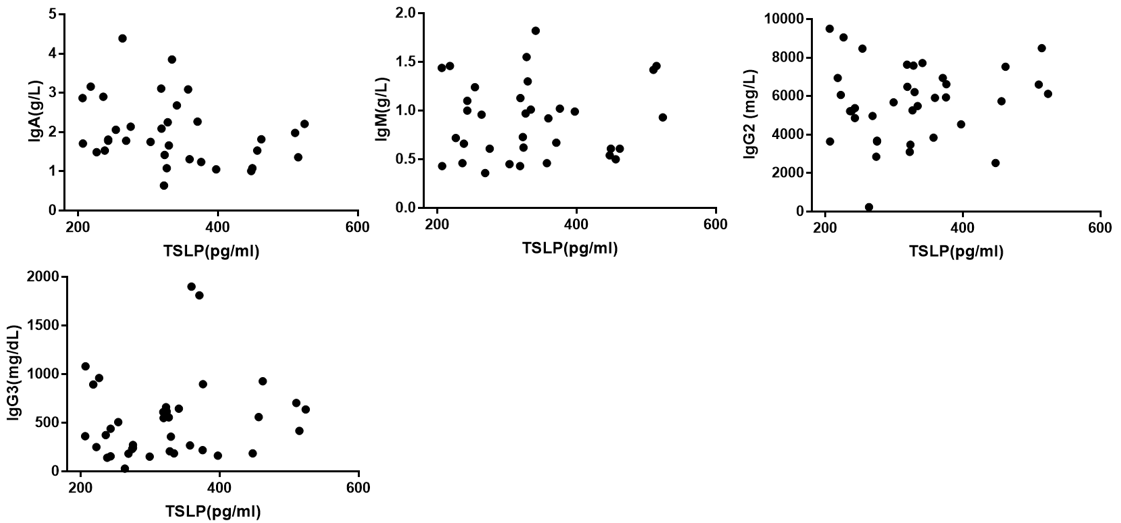


Figure S5. TSLP could not polarize Naïve T cells to Tfh directly.


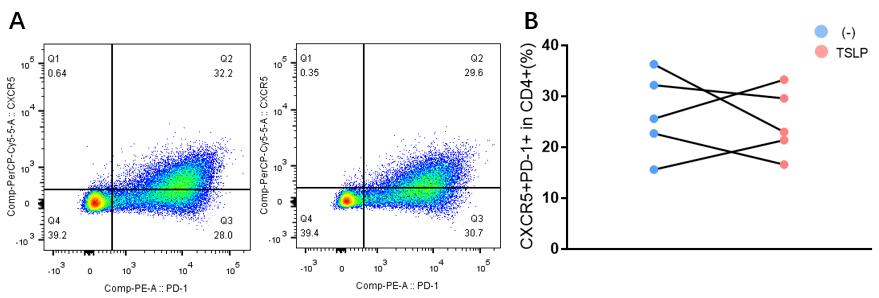


CD4+ Naïve T cells from HC were stimulated with anti-CD3, anti-CD28, with or without TSLP, and the percentages of Tfh were detected on 5 days. (A) Representative FACS of Tfh, with (right) or without (left) TSLP. (B) Summary graph of the percentages of Tfh between the two groups.

Figure S6. The percentage of Tfh was positively correlated with the percentages of OX40L+ on B cells in the peripheral blood of patients with IgG4-RD.


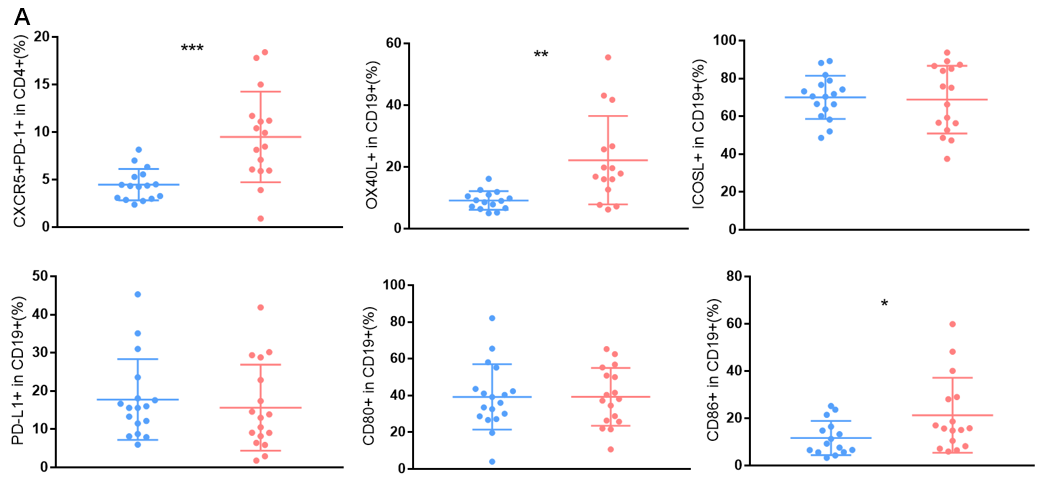


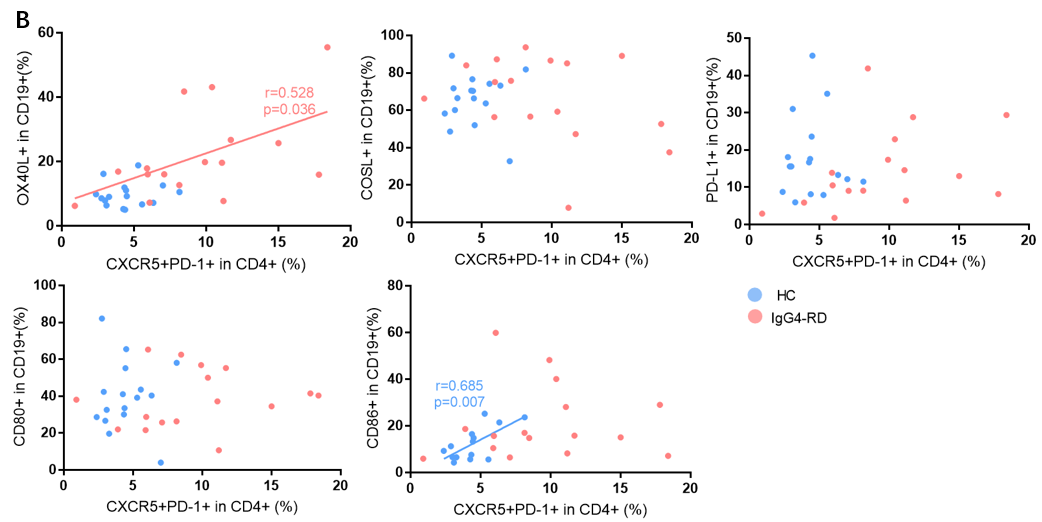


(A) The percentages of Tfh in CD4+ T cells and co-stimulatory molecules on B cells in HC and IgG4-RD patients. (B) Correlations between the percentage of Tfh and co-stimulatory molecules on B cells. *=p<0.05; **=p<0.01; ***=p<0.001.

Figure S7. Representative western blot showed the phosphorylation of JAK-STAT family with different time.


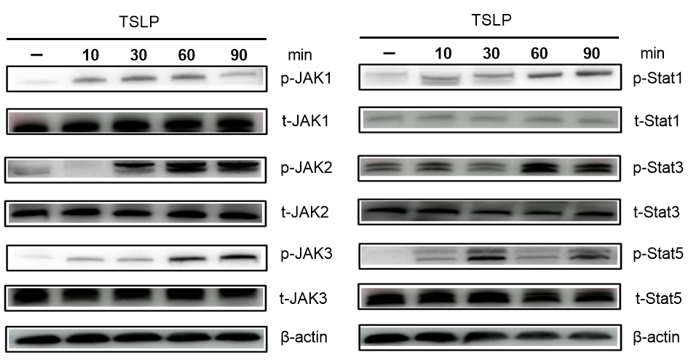


B cells was pre-activated with anti-IgM, CD40L for 72h, and then stimulated with TSLP for 0, 10, 30, 60, 90 minutes. Phosphorylation of JAK-STAT family was detected by western blot.

Figure S8. Weight, imaging findings and TSLP expression of Lat mice.


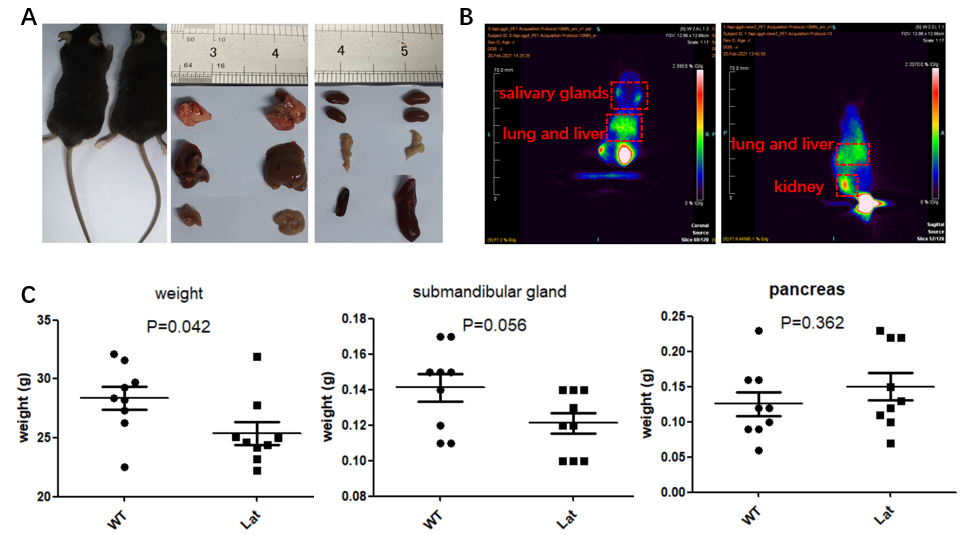


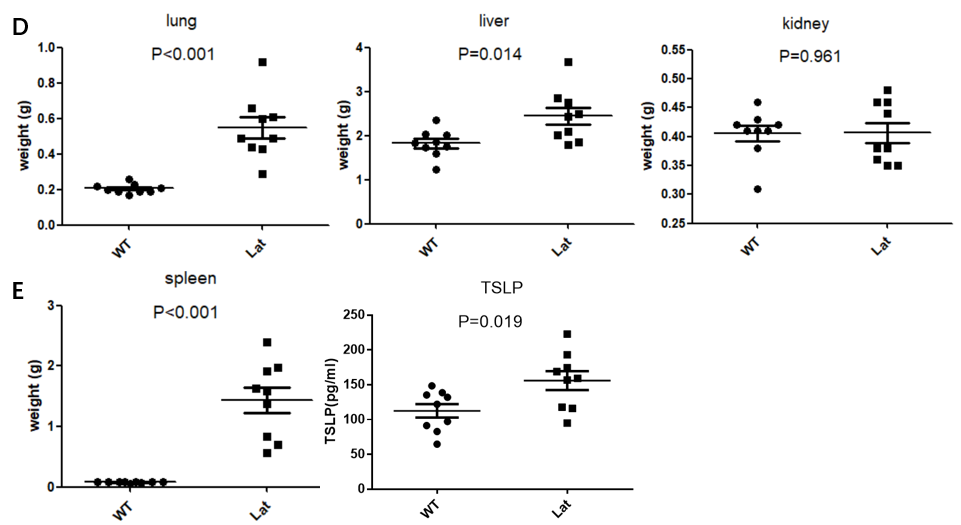


(A) Lung, liver, submandibular glands (SMGs), kidneys, pancreas, and spleen of the WT (left) and Lat mice (right) at 12 weeks old. (B) PET-CT showed the involved organs of a Lat mice. (C-E) Comparison of body weight, the weight of SMGs, pancreas, lung, liver, kidney, spleen, and the plasma level of TSLP between the WT (n=9) and Lat mice (n=9).

Figure S9. Immunohistochemistry of IgG1 in affected organs of Lat mice.


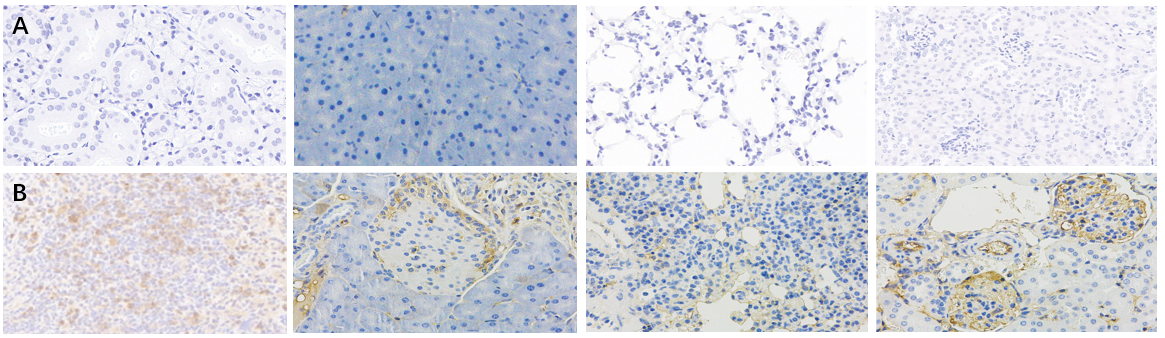


Immunohistochemistry (200X) showed IgG1 staining of SMGs, pancreas, lung, and kidneys from representative WT (A) and Lat mice (B).

Figure S10. Expressions TSLP and TSLPR in affected organs of Lat mice.


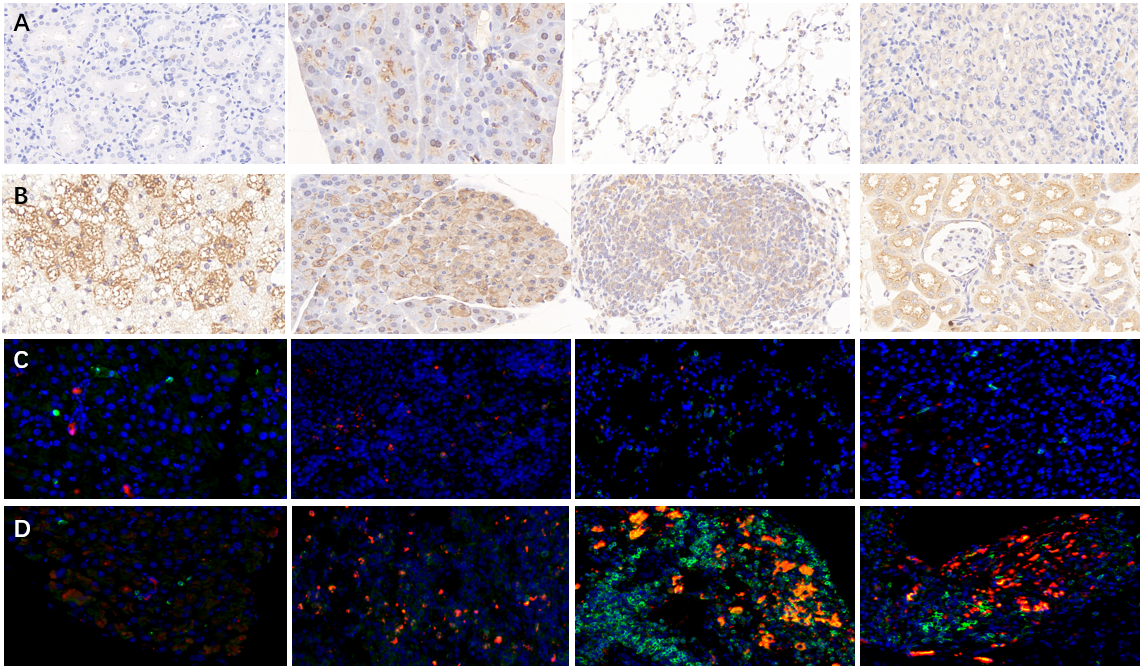


Immunohistochemistry (200X) showed TSLP staining of submandibular glands (SMGs), pancreas, lung, and kidneys from representative WT (A) and Lat mice (B). Immunofluorescence displayed the colocalization of B220 (green) and CRLF2 (TSLPR, red) in pancreas, spleen, lung, and kidneys from representative WT (C) and Lat mice (D).

Figure S11. Application of different dosage of anti-TSLP antibody Lat mice.


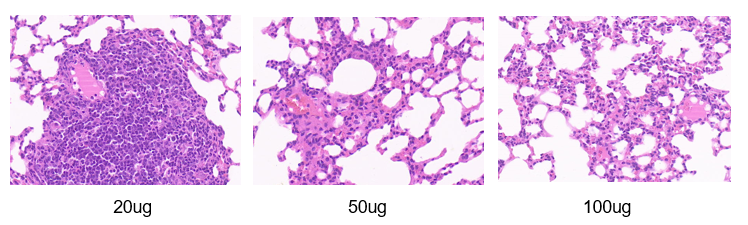


Representative H&E-stained sections (100X) of inflammation area in the lung tissue of Lat mice with different dosage of anti-TSLP therapy: 20ug (n=2), 50ug (n=2), and 100ug (n=2).
