# Supplementary material for: TSLP promoting B cell proliferation and polarizing follicular helper T cell as a therapeutic target in IgG4-related disease
Source: J Transl Med. 2022 Sep 8;20:414. doi: 10.1186/s12967-022-03606-1 (PMC9461269; doi:10.1186/s12967-022-03606-1)
Supplement: Supplementary file 2 — Additional file 2: Table S1. Baseline demographic features, clinical characteristics, and laboratory parameters of 71 treatment naïve patients with IgG4-RD. [file 12967_2022_3606_MOESM2_ESM.docx]

Table S1. Baseline demographic features, clinical characteristics, and laboratory parameters of 71 treatment naïve patients with IgG4-RD.

| Variables | Patients (n=71) |
| --- | --- |
| Male gender, n (%) | 37(52.1) |
| Age (years), M (Q1-Q3) | 56(49-62) |
| Disease duration (years), M (Q1-Q3) | 1.0(0.4-2.3) |
| *Symptoms at disease onset (n, %)* |  |
| Lacrimal gland enlargement | 26(36.6) |
| Submandibular gland enlargement | 21(29.6) |
| Parotid gland enlargement | 5(7.0) |
| Abdominal pain | 9(12.7) |
| Nausea and vomiting | 2(2.8) |
| Nasal congestion | 5(7.0) |
| Jaundice | 4(5.6) |
| Itching | 4(5.6) |
| Cough | 3(4.2) |
| Lymph node swelling | 15(21.1) |
| Back pain | 4(5.6) |
| Arthralgia | 2(2.8) |
| *Organ affected (n, %)* |  |
| Submandibular gland | 39(54.9) |
| Lacrimal gland | 27(38.0) |
| Parotid gland | 11(15.5) |
| Pancreas | 22(31.0) |
| Bile duct | 6(8.5) |
| Lymph node | 28(39.4) |
| Pituitary | 2(2.8) |
| Sinus | 15(21.1) |
| Thyroid gland | 2(2.8) |
| Lung | 11(15.5) |
| Liver | 2(2.8) |
| Kidney | 8(11.3) |
| Prostate | 5(7.0) |
| Aortitis/periaortitis | 8(11.3) |
| Skin | 2(2.8) |
| *Laboratory parameters* |  |
| HGB (g/L), mean±SD | 133.0±20.5 |
| WBC (109/L), mean±SD | 6.89±2.22 |
| PLT (109/L), mean±SD | 248.7±71.6 |
| EOS (109/L), mean±SD | 0.53±0.14 |
| ESR (mm/h), M (Q1-Q3) | 16(8-33) |
| hsCRP(mg/L), M (Q1-Q3) | 1.22(0.35-3.61) |
| IgG(g/L), mean±SD | 21.9±9.5 |
| IgA (g/L), mean±SD | 2.00±0.81 |
| IgM(g/L), mean±SD | 0.88±0.48 |
| IgG1 (mg/L), M (Q1-Q3) | 8895(7715-10925) |
| IgG2 (mg/L), M (Q1-Q3) | 5755(4627-7395) |
| IgG3 (mg/L), M (Q1-Q3) | 500(230-780) |
| IgG4 (mg/L), M (Q1-Q3) | 11100(4367-19650) |
| T-IgE (KU/L), M (Q1-Q3) | 406(152-972) |
| RI | 8(4-10) |

M, median; Q1, quartile 1; Q3, quartile 3; WBC, white blood cell; HGB, hemoglobin; PLT, platelet; EOS, eosinophils; ESR, erythrocyte sedimentation rate; hsCRP, hypersensitive C-reactive protein; Ig, immunoglobulin; T-Ig, total immunoglobulin; RI, responder index.
