# Supplementary material for: TSLP promoting B cell proliferation and polarizing follicular helper T cell as a therapeutic target in IgG4-related disease
Source: J Transl Med. 2022 Sep 8;20:414. doi: 10.1186/s12967-022-03606-1 (PMC9461269; doi:10.1186/s12967-022-03606-1)
Supplement: Supplementary file 4 — Additional file 4: Supplementary Methods. [file 12967_2022_3606_MOESM4_ESM.docx]

Additional Materials

Additional Methods

RNA quantification and qualification

RNA was extracted using Trizol (Invitrogen) according to the manufacturer’s

instruction.Total amounts and integrity of RNA were assessed using the RNA Nano 6000 Assay Kit of the Bioanalyzer 2100 system (Agilent Technologies, CA, USA).

Library preparation

A total of 2 μg RNA per sample was used for library preparations. Sequencing

libraries were generated using NEBNext UltraTM RNA Library Prep Kit for Illumina (NEB) following the manufacturer’s recommendations and index codes were added to attribute sequences to each sample. PCR library was purified and library quality was assessed on the Agilent Bioanalyzer 2100 system.

Clustering and sequencing

After the library is qualified, the different libraries are pooling according to the

effective concentration and the target amount of data off the machine, then being sequenced by the Illumina NovaSeq 6000. The end reading of 150bp pairing is generated. The basic principle of sequencing is to synthesize and sequence at the same time (Sequencing by Synthesis). Four fluorescent labeled dNTP, DNA polymerase and splice primers were added to the sequenced flowcell and amplified. When the sequence cluster extends the complementary chain, each dNTP labeled by fluorescence can release the corresponding fluorescence. The sequencer captures the fluorescence signal and converts the optical signal into the sequencing peak by computer software, so as to obtain the sequence information of the fragment to be tested.

Quality control

The image data measured by the high-throughput sequencer are converted into

sequence data (reads) by CASAVA base recognition. Raw data (raw reads) of fastq format were firstly processed through in-house perl scripts. In this step, clean data (clean reads) were obtained by removing reads containing adapter, reads containing N base and low quality reads from raw data. At the same time, Q20, Q30 and GC content the clean data were calculated. All the downstream analyses were based on the clean data with high quality respectively.

Reads mapping to the reference genome

Reference genome and gene model annotation files were downloaded from

genome website directly. Index of the reference genome was built using Hisat2

(v2.0.5) and paired-end clean reads were aligned to the reference genome using Hisat2 (v2.0.5). We selected Hisat2 as the mapping tool for that Hisat2 can generate a database of splice junctions based on the gene model annotation file and thus a better mapping result than other non-splice mapping tools.

Quantification of gene expression level

featureCounts (v1.5.0-p3) was used to count the reads numbers mapped to each gene. And then FPKM of each gene was calculated based on the length of the gene and reads count mapped to this gene. FPKM, expected number of Fragments Per Kilobase of transcript sequence per Millions base pairs sequenced, considers the effect of sequencing depth and gene length for the reads count at the same time, and is currently the most commonly used method for estimating gene expression levels.

Differential expression analysis

Differential expression analysis of two conditions/groups (two biological replicates per condition) was performed using the DESeq2 R package (1.20.0). DESeq2 provide statistical routines for determining differential expression in digital gene expression data using a model based on the negative binomial distribution. The resulting P-values were adjusted using the Benjamini and Hochberg’s approach for controlling the false discovery rate. padj<=0.05 and |log2(foldchange)| >= 1 were set as the threshold for significantly differential expression.

Enrichment analysis of differentially expressed genes

KEGG is a database resource for understanding high-level functions and utilities of the biological system, such as the cell, the organism and the ecosystem, from molecular-level information, especially large-scale molecular datasets generated by genome sequencing and other high-through put experimental technologies (<http://www.genome.jp/kegg/>). We used clusterProfiler R package (3.8.1) to test the statistical enrichment of differential

expression genes in KEGG pathways.
